# Synergistic Treatment of Congo Red Dye with Heat Treated Low Rank Coal and Micro-Nano Bubbles

**DOI:** 10.3390/molecules27134121

**Published:** 2022-06-27

**Authors:** Ning Han, Rong Cui, Haisen Peng, Ruize Gao, Qiongqiong He, Zhenyong Miao

**Affiliations:** 1 National Engineering Research Center of Coal Preparation and Purification, China University of Mining and Technology, Xuzhou 221008, China; 15651462381@163.com (N.H.); cr18734598390@163.com (R.C.); wxtphs1314@163.com (H.P.); ruize.gao@cumt.edu.cn (R.G.); 2 School of Chemical Engineering and Technology, China University of Mining and Technology, Xuzhou 221008, China

**Keywords:** low-rank coal, semi-coke, micro-nano bubbles, Congo red, synergistic

## Abstract

In this study, the adsorption method and micro-nano bubble (MNB) technology were combined to improve the efficiency of organic pollutant removal from dye wastewater. The adsorption properties of Congo red (CR) on raw coal and semi-coke (SC) with and without MNBs were studied. The mesoporosity of the coal strongly increased after the heat treatment, which was conducive to the adsorption of macromolecular organics, such as CR, and the specific surface area increased greatly from 2.787 m^2^/g to 80.512 m^2^/g. MNBs could improve the adsorption of both raw coal and SC under different pH levels, temperatures and dosages. With the use of MNBs, the adsorption capacity of SC reached 169.49 mg/g, which was much larger than that of the raw coal at 15.75 mg/g. The MNBs effectively reduced the adsorption time from 240 to 20 min. In addition, the MNBs could ensure the adsorbent maintained a good adsorption effect across a wide pH range. The removal rate was above 90% in an acidic environment and above 70% in an alkaline environment. MBs can effectively improve the rate of adsorption of pollutants by adsorbents. SC was obtained from low-rank coal through a rapid one-step heating treatment and was used as a kind of cheap adsorbent. The method is thus simple and easy to implement in the industrial context and has the potential for industrial promotion.

## 1. Introduction

In recent years, a large amount of dyeing and printing wastewater has been produced with the success of the dyeing and printing industry. According to statistics, more than 10,000 tons of dye are used every year around the world [1]. It is estimated that 2% of the dye is directly discharged into wastewater every year, causing great harm to the environment [2]. The composition of printing and dyeing wastewater is complex, containing a large number of dye contaminants, heavy metals, dye carriers, surfactants, dispersants and other organic additives [3,4]. Therefore, the treatment of dyeing wastewater is an urgent problem that needs to be solved. At present, dyeing and printing wastewater treatment methods include the flocculation method [5], adsorption method [6,7,8,9], membrane infiltration method [10], oxidation method [11] and biological method [12]. Maruthupandy [13] used photocatalytic degradation of CR and achieved 98% degradation at 40 min. Liang [14] prepared a protein super-sorbent and the mechanism of adsorption was explained in detail. The adsorption method has been widely used because of its advantages, such as a simple process, good effect and lack of secondary pollution [15]. Activated carbon is used in the dye adsorption method, but its price is relatively high [16]. The preparation of cheap adsorbents is the premise of industrialization [17].

The reserves of low-rank coal are enormous, accounting for about half of the world’s discovered coal resources [18]. At present, low-rank coal has low calorific values and a low utilization rate. Low-rank coal has a porous structure [19], and there are abundant carboxylic acid and phenolic hydroxyl groups on the surface [20], which provide a large number of adsorption sites for the removal of CR [21]. Zhang [22] used lignite to treat phenolic wastewater. Mohan [23] used lignite to remove heavy metal ions from mine wastewater. He [24] used a lignite-based load for adsorption dye. All these studies demonstrated the good adsorption performance of low-rank coal.

Microbubbles (MBs) and nanobubbles (NBs) are bubbles with diameters of 10–50 μm and <200 nm, respectively [25]. NBs have characteristics including a large specific surface area, negative charge on the surface, long residence time in water, high mass transfer efficiency and abundant active free radicals, giving them broad application prospects [26,27]. Micro-nano bubbles (MNBs) exhibit a strong ability to decompose various organic pollutants in wastewater [28]. Liu [29] found that, under the same conditions, the effect of MNBs in the coagulation flotation process for dyeing and printing wastewater was 30% stronger than that of conventional bubbles.

In this study, we combined adsorption and oxidation methods to work synergistically with SC MNBs. The MNBs were used to bring the contaminants to the surface of the SC and achieve effective removal of CR dye. Low-rank coal and its SC were used as cheap adsorbents, and the adsorption performance (adsorption isotherm and thermodynamics, adsorption kinetics, dosage, pH) of the CR resulting from the synergistic effect of raw coal, semi-coke and MNBs was studied. The adsorption capacity was studied in detail and adsorption behavior was evaluated and compared with other adsorption methods.

## 2. Materials and Instruments

### 2.1. Reagent Preparation

The low-rank coal sample was Wanli long flame coal from Hongqinghe Coal Mine in Yitai. The heat treatment temperature was 900 °C and the treatment time was 8 min, following our previous results [30]. CR reagent (C_32_H_22_N_6_Na_2_O_6_S_2_) was purchased from Nanjing Chemical Reagent Co. Ltd. (Nanjing, China). H_2_SO_4_ and NaOH were purchased from Sinopharm Chemical Reagents Co. Ltd. (Shanghai, China). All chemicals were of analytical grade and used as received.

### 2.2. Preparation of SC

The coal samples were crushed in a hammer crusher and then sieved to make sure the particle sizes of the samples were less than 0.5 mm. Then, the heat treatment experiment with the diameter less than 0.5 mm long-flame coal was carried out in a 900 °C tubular furnace over 8 min.

### 2.3. Adsorption Experiments

In order to study the effect of the pH value of the solution on the adsorption of CR dye, CR solution with an initial concentration of 100 mg/L was selected, and the pH was regulated by 0.1 M H_2_SO_4_ and NaOH solutions. The adsorption kinetics experiment was performed at 25 °C by adding 0.3 g of adsorbent to 50 mL of solutions containing 100 mg/L of CR dye. Then, the solids and remaining solution were separated using a centrifuge.

The dye concentration of the CR was determined using an ultraviolet-visible (UV) spectrophotometer. In order to measure the adsorption effect of the low-rank coal and its SC on the CR dye, the percentage removal rate of the CR dye (γ) and the adsorption amount (q) were used as the evaluation indexes for the adsorption effect. The residual concentration of the CR dye solution was calculated by substituting the absorbance value measured by the UV spectrophotometer into the standard working curve of CR. γ and q can be calculated according to the following formula:

(1) $$\gamma =\frac{C_{0}-C_{t}}{C_{0}}\times 100\%$$

(2) $$q=\frac{V\left(C_{0}-C_{t}\right)}{m}$$

where C_0_ (mg/L) and C_t_ (mg/L) are the initial and equilibrium concentration of the dye respectively, m (g) is the mass of adsorbent and V (L) is the amount of dye solution.

### 2.4. Sample Characterization

The topography and microstructure of raw coal and SC were observed using scanning electron microscopy (Sigma, Neustadt, Germany) and an automatic specific surface nitrogen adsorption instrument (BEL-MAX, Osaka, Japan). The CR concentration was measured with a UV spectrophotometer (4802s UV/VIS, Shanghai). Fourier-transform infrared (FTIR) spectra were obtained with a FTIR spectrometer (Bruker VERTEX, Ettlingen, Germany). The MNB generating device was purchased from Xiazhichun Environmental Protection Technology Co. Ltd. (XZCP-K-0.75, Chongqing, China). 

## 3. Results and Discussion

### 3.1. The Characterization of Raw Coal and SC

**Figure 1:**
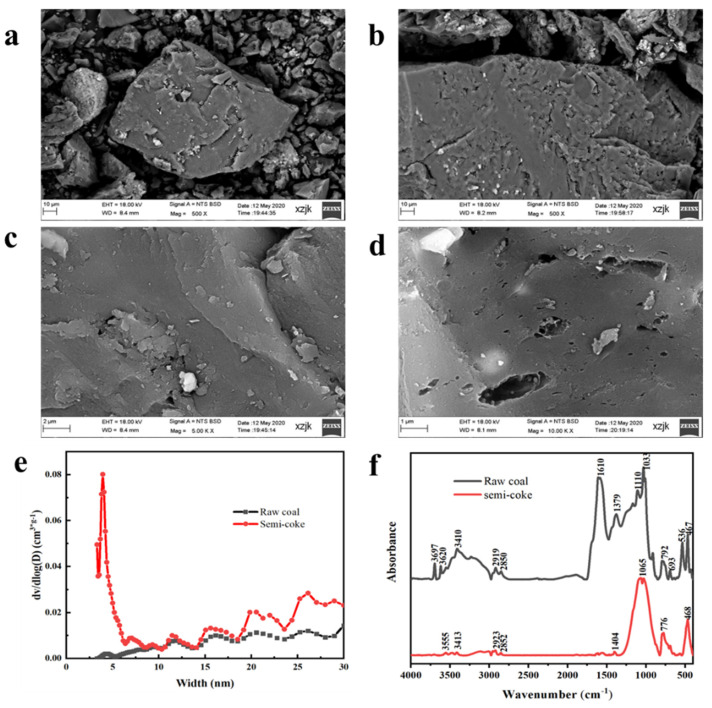
SEM images of the raw coal (**a**,**c**) and the SC (**b**,**d**); the pore size distribution of raw coal and SC (**e**); FTIR spectra of raw coal and SC (**f**).

As can be seen from Figure 1a,b, the surface of the raw coal was smooth on the whole, with a small number of pores. Compared with the raw coal, the surface morphology and internal structure of the SC changed to some extent, and the residual moisture and volatile content of the SC decreased after heat treatment, which promoted the development of new pores. This corresponds with results from a previous study, which found that pore development in low-rank coke was caused by the cracking of oxygen-containing functional groups during pyrolysis [31]. However, the ash and fixed carbon content in the SC were higher than that of the raw coal. Therefore, more pores appeared on the surface of the coal sample, and the surface morphology changed obviously. These pores provided adsorption sites and transport channels for the CR. 

In conformity with Figure 1a,b, the microporous content of the raw coal was low, increasing sharply after heat treatment at 900 °C, and the SC had a large number of micropores. Furthermore, micropores were the main influencing factor for the specific surface area. Compared with the raw coal, the specific surface area of the SC increased significantly. This change resulted in a more favorable impact on the removal of CR. Moreover, the number of mesopores in the SC increased significantly. Some studies have found that low-rank coal has a strong adsorption capacity for cyclic organic matter, with these pores providing sites of attachment for solution adsorption [32].

The specific surface area and pore size were analyzed using nitrogen adsorption and desorption, and the results were calculated using the Brunauer-Emmett-Teller (BET) method and the Barrett-Joyner-Halenda (BJH) method [33]. The specific surface area markedly increased from 2.787 m^2^/g in raw coal to 80.512 m^2^/g in SC. The volatile matter of the coal was released at 900 °C, which opened a large number of pores and expanded the pore structure, resulting in an increase in the specific surface area and total pore volume. This was because, at very high temperatures (900 °C), part of the pore structure collapses and forms micropores.

The oxygen-containing functional groups of the raw coal and SC were obtained using an FTIR spectrometer, as shown in Figure 1d. The peaks at 3700–3200 cm^−1^ were attributed to the O–H vibration. The peaks appearing near 3000–2800 cm^−1^ were saturated aliphatic hydrocarbon vibration peaks. The peaks at 1610–1580 cm^−1^ were a C=C stretching vibration peak and C=O expansion and the peak near 1697 cm^−1^ was attributed to contraction vibration. The C–O stretching vibration peak (1100–1000 cm^−1^) could also be found in the FTIR spectrum [34,35].

When the raw coal was treated at 900 °C, the degrees stretching vibration for O-H (3700–3200 cm^−1^), C-H (3000–2800 cm^−1^) and C=O (1697 cm^−1^) were weakened to a certain extent. This can be attributed to the fact that the high temperature of 900 °C completely destroyed the structure of the aliphatic hydrocarbons in the raw coal and released them. In contrast to before heating, C=C (1610–1580 cm^−1^) did not appear. This can be explained by the fact that the C=C group in the raw coal sample showed infrared activity and the symmetry was distorted by the adjacent C=O and C-O species. When samples are pyrolyzed at the high temperature of 900 °C, adjacent C=O and C–O species are severely destroyed [36]. Therefore, no C=C vibration peak was found in the infrared spectrum of the SC. The raw coal and SC spectra at 1100–1000 cm^−1^ were attributed to the stretching vibration peak of C-O because the C-O group was relatively stable [37]. Stretching vibration peaks at 536 cm^−1^ and 693 cm^−1^ are characteristic of aromatic disulfide, indicating organic sulfur (-S-S-) in the raw coal. After heat treatment at 900 °C, these two peaks also disappeared, indicating decomposition of sulfur ether, which was consistent with previous research results [38]. In addition, other studies [39] have also suggested that oxygen-containing functional groups play an important role in the adsorption process in solutions. These results suggest that many oxygen-containing functional groups in the sample itself decomposed during the heat treatment. At the same time, when the MNBs collided with the SC particles in the solution, the gas–liquid and gas–solid connections were capillary bridge connections [40]. This type of contact can help stabilize the bubbles [41]. When bubbles burst, a large amount of hydroxyl radicals are produced [42], which exert a strong oxidation effect. The MNBs were negatively charged, and the double electric layers were stable. Thus, they repelled each other, prevented bubbles from merging and reduced the rupture rates. The stagnation time in water was longer, which could have further increased the removal efficiency.

### 3.2. Influence of pH and Time on Adsorption with or without Bubbles

**Figure 2:**
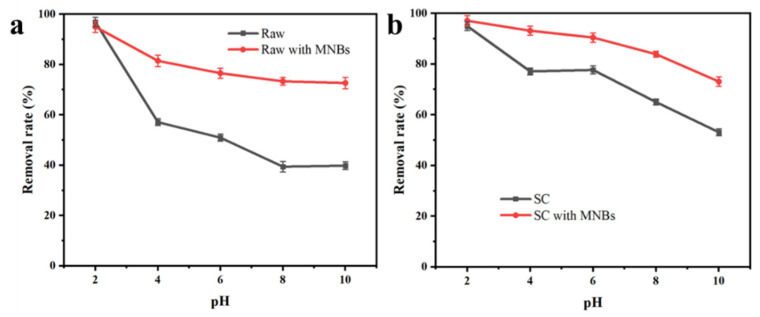
Influence of pH value on adsorption of CR (**a**) dye by raw coal and SC (**b**) with and without bubbles.

The influence of pH value on the adsorption of CR dye by raw coal and SC with and without bubbles is shown in Figure 2. The CR removal rates for coal and semi-coke with MNBs were much higher than without MNBs, and the differences increased under alkaline conditions. As shown in Figure 2b, the SC with MNBs had a stronger adsorption capacity for CR, with a removal rate higher than 90% under the condition of pH < 6, and even in an alkaline environment the CR removal rate of SC was still above 70%. In the presence of MNBs, the removal effect changed little with changes in the pH value, which meant the MNBs could alleviate the negative effect of pH on the removal effect.

The removal rate decreased with the increase in pH for raw coal and SC with and without MNBs. A much better CR removal effect with raw coal and SC was obtained under acidic conditions than that under neutral and alkaline conditions. This was because CR is negatively charged at a low pH value [43], and the carboxyl and hydroxyl groups carried by the adsorbent itself were protonated (-COOH_2_^+^ and -OH_2_^+^). The number of positively charged sites in the adsorbent gradually increased, and the electrostatic interaction between the adsorbent and the negatively charged CR enhanced their adsorption, which was one of the reasons for the removal of CR. At high pH values, the carboxyl and hydroxyl groups on the adsorbent surface were deprotonated (-COO- and -O-), and electrostatic repulsion between the adsorbent and CR dye resulted in a low removal rate for the CR. Industrially, adjusting the pH to acidic would lead to an increase in adsorption costs, and subsequent adjustment of the pH back to neutral would lead to an increase in the salt content of the wastewater. The MNBs enabled the adsorbent to retain a large adsorption capacity in both neutral and alkaline environment, which is beneficial for the use of adsorbents.

### 3.3. Effect of Adsorbent Dosage

**Figure 3:**
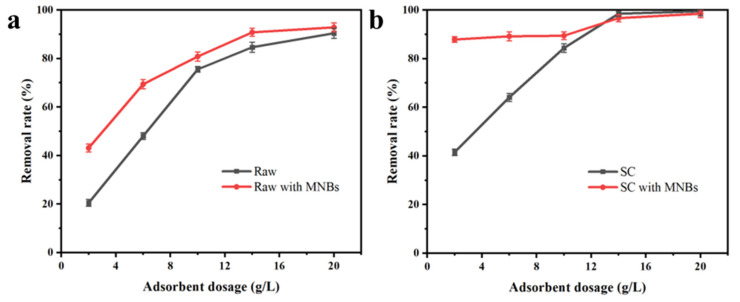
The CR removal rates for (**a**) raw coal and (**b**) SC with different adsorbent dosages.

The effect of adsorbent dosage on adsorption is shown in Figure 3. The removal rate of the SC without MNBs (41.5% at 2 g/L adsorbent dosage) was much higher than that of the raw coal (20.7% at 2 g/L adsorbent dosage) due to the higher porosity of the SC caused by the pyrolysis. When MNBs were used, as we can see from Figure 3a, the removal rate showed a great difference when the dosage was 2 g/L. The difference between the raw samples with MNBs (43.11%) and the raw samples without MNBs (20.5%) was about 22.61%. This was partly due to the free radicals generated by the collapse of MNBs, and about 20% of the CR was degraded by free radicals. High CR removal by SC could be achieved with a small amount of adsorbent (87.88% at 2 g/L adsorbent dosage), as seen from Figure 3b. MNBs act as bridges, carrying pollutants to the surface of the adsorbent, which is another reason for the improvement in adsorption. It can be seen from the above that, whether it was raw coal or SC, a better dye removal effect could always be achieved in the presence of MNBs. 

### 3.4. Kinetic Studies of CR Dye Adsorption

The results for the adsorption kinetics were fitted with a pseudo-first-order model and pseudo-second-order model [44]. The following formulas were used:

Lagergren pseudo-first-order adsorption rate equation:

(3) $$\ln\left(q_{e}-q_{t}\right)=\ln q_{e}-k_{1}t$$

Lagergren pseudo-second-order adsorption rate equation:

(4) $$\frac{t}{q_{t}}=\frac{1}{k_{2}q_{e}{}^{2}}+\frac{t}{q_{e}}$$

where t (min) is the adsorption time, q_t_ (mg/g) is the adsorption capacity at time t, q_e_ (mg/g) is the equilibrium adsorption capacity, k_1_ (min^−1^) is the pseudo-first-order adsorption rate constant, and k_2_ (g/(mg·min)) is the pseudo-second-order adsorption rate constant.

**Figure 4:**
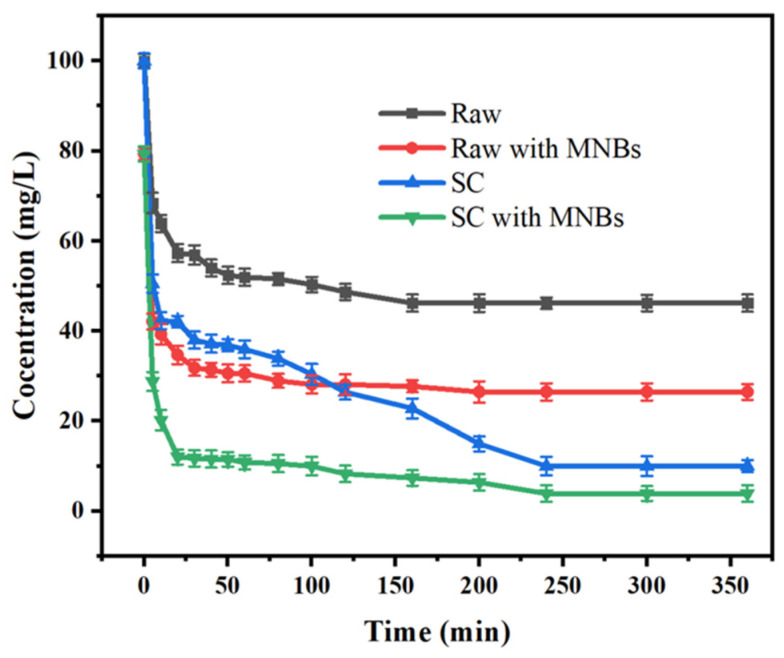
Influence of adsorption time on concentration and adsorption capacity with and without bubbles.

The adsorption behavior of the CR on the raw coal and SC in terms of the adsorption time was investigated. As shown in Figure 4, MNBs accelerated the removal rate at the early stage of adsorption, and the removal rate reached 88% in 20 min for the SC with MNBs, while it took more than 50 min to obtain the same removal rate for the SC without MNBs. The adsorption equilibrium time for the raw coal without MNBs was 120 min, and it took about 10 min to reach the same residual CR concentration with MNBs. This result was attributed to the increase in the carbonyl content. This was because the MNB rupture process produced -OH, enhancing the oxidation capacity of the system. While accelerating the mass transfer efficiency of the CR degradation process, CR further diffused into the solution and increased dispersion, the -OH and CR molecule contact probability increased and the response was more adequate. In addition, under the conditions of very high temperature and pressure inside the MNBs, the dye was further pyrolyzed, resulting in an increase in the rate of degradation. Therefore, the adsorption time needed for the CR solution to reach a residual concentration was markedly lower than that without MNBs for the same concentration, indicating that the combination of micro-nano bubble technology and the adsorption method could effectively reduce the adsorption time.

**Figure 5:**
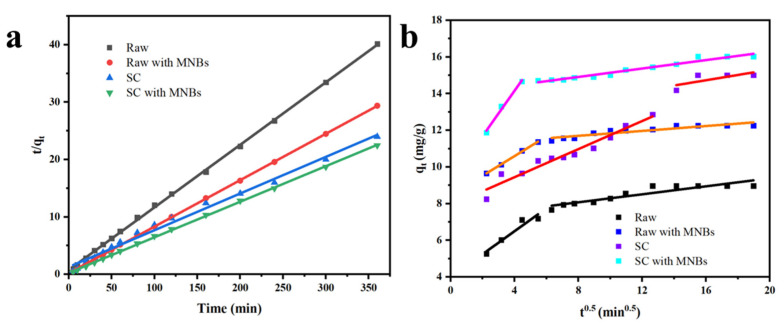
Pseudo-second-order for dye adsorption onto raw coal and SC (**a**). Fitting curves for internal diffusion of SC adsorbing CR dye (**b**).

The linear fitting results for the adsorption of CR dye on SC with and without MNBs are shown in Figure 5. The relevant kinetic parameters and correlation coefficients are summarized in Table 1. The pseudo-second-order model (R^2^ = 0.9996) fitted the experimental data better than pseudo-first-order kinetic model (R^2^ = 0.8106), which meant the adsorption rate of CR was controlled by chemisorption [45,46]. Additionally, the theoretical adsorption capacity of the SC with MNBs was 16.14 mg/g (Table 1), which was little different from the experimental data that gave 16.02 mg/g. 

To further study the mass transfer behavior in the process of solid–liquid adsorption, the Weber–Morris internal diffusion equation was used [47]. The formula is as follows:

(5) $$q_{t}=K_{d}t^{1/2}+I$$

where K_d_ (mg/(g · g.min^1/2^)) is the diffusion rate constant in particles and I is a constant related to the thickness of the adsorption layer and boundary layer. 

Fitting curves of t^1/2^ and q_t_ with the internal diffusion model for the raw coal and SC adsorbing CR dye are shown in Figure 5b. The curves do not transect the base point, which indicates that the process of internal diffusion during the adsorption was not controlled by a single rate. At the beginning of adsorption, CR dye molecules diffused rapidly to the surface of the adsorbent from the solution. Then, the dye molecules diffused to the pores and the final slope was almost zero, indicating that the adsorption process tended towards an equilibrium. Therefore, the adsorption rates at different stages of the adsorption process were affected by different factors.

### 3.5. Adsorption Isotherms

The Langmuir and Freundlich isotherm models were produced according to Equations (6) and (7) [48], respectively.

The Langmuir adsorption isotherm equation is:

(6) $$\frac{C_{e}}{q_{e}}=\frac{1}{{\mathrm{bq}}_{0}}+\frac{C_{e}}{q_{0}}$$

The Freundlich adsorption isotherm equation is:

(7) $$\ln q_{e}=\ln k+\frac{1}{n}\ln C_{e}$$

where C_e_ (mg/L) represents the adsorption equilibrium concentration; q_e_ (mg/g) represents the equilibrium adsorption capacity; q_0_ (mg/g) represents the saturated adsorption capacity of a single molecule; and b, k and n represent adsorption equilibrium constants.

**Figure 6:**
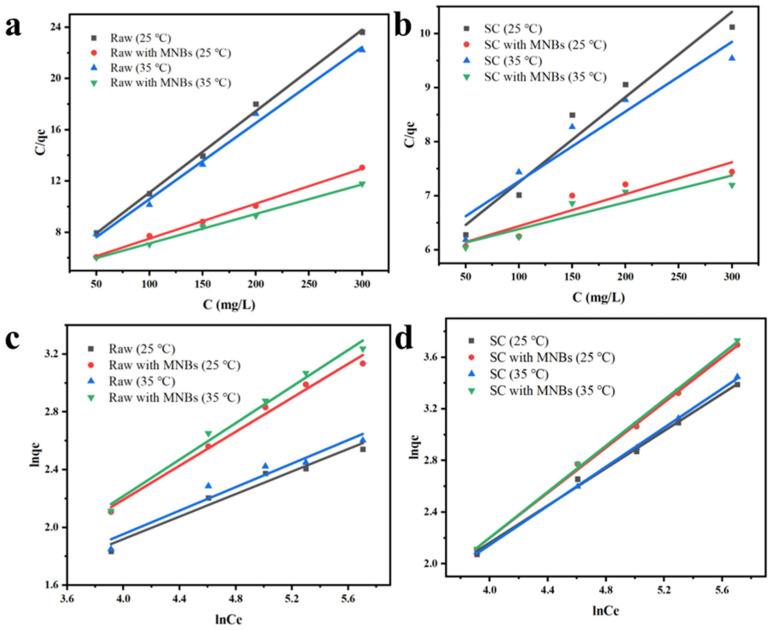
The Langmuir model for raw coal and SC with and without bubbles at different temperatures (**a**,**b**); the Freundlich model for raw coal and SC (**c**,**d**).

Adsorption experiments with different initial concentrations in the range of 50–300 mg L^−1^ of CR were performed at 25 °C and 35 °C over 2 h. The experimental data for the CR dye adsorption were fitted with the Langmuir and Freundlich adsorption isotherm equations, as shown in Figure 6. Based on the fitting results for the adsorption isotherm models, the thermodynamic parameters are shown in Table 2.

The isotherm models were fitted to the experimental data, and the equilibrium maximum adsorption amounts and associated fitting coefficients for raw coal, raw coal with MNBs, SC and SC with MNBs are listed in Table 2. As shown in Figure 6, when the temperatures were 25 °C and 35 °C, the Langmuir isotherm model described the adsorption data better than the Freundlich model, indicating that the Langmuir model fit better described the adsorption with raw coal and raw coal with MNBs, and the adsorption isotherms were suitable for monolayer adsorption [49,50]. Moreover, the maximum adsorption capacities of raw coal, raw coal with MNBs, SC and SC with MNBs were 15.75 mg g^−1^, 36.64 mg g^−1^, 63.45 mg g^−1^ and 169.49 mg g^−1^ at 25 °C, respectively. The adsorption capacity of SC with MNBs was much higher than those of the others. This was mainly attributed to the synergistic mechanism of the SC with the MNBs. At 35 °C, the maximum adsorption capacity was 201.61 mg g^−1^. The results showed that increasing the temperature was beneficial to the reaction, and the reaction was endothermic. For SC and SC with MNBs, the Freundlich isotherm model described the adsorption data better than the Langmuir model, indicating that the Freundlich model fit better described the adsorption of CR on SC, which was considered to be a multilayer adsorption process. 

A comparison of the adsorption of CR by SC with MNBs and other adsorbents is presented in Table 3. All these results show that the SC with MNBs method we used was effective in achieving the removal of CR. The adsorption capacity of SC with MNBs was found to be greater than that of some previously reported adsorbents and, in addition, the time needed to reach equilibrium was greatly reduced.

## 4. Conclusions

Low-rank coal and its SC were used as cheap adsorbents for dye adsorption, and MNBs were used to improve the adsorption. With MNBs, the adsorption capacity of SC reached 169.49 mg/g, which was much larger than that of the raw coal at 15.75 mg/g. MNBs accelerated the removal rate at the early stage of adsorption and effectively reduced the adsorption time, and the removal rate reached 88% in 20 min for SC with MNBs, while it took more than 50 min to obtain the same removal rate for SC without MNBs. In addition, MNBs could ensure the adsorbent maintained a good adsorption effect across a wide pH range. The removal rate was above 90% in an acidic environment and above 70% in an alkaline environment. The experimental data for the adsorption kinetics and thermodynamics could be well-described with a pseudo-second-order model and Freundlich model. The improvement effect of micro-nano bubbles on adsorption also has important reference significance for the adsorption of other adsorbents.

## Figures and Tables

**Table 1:** Kinetic parameters for CR dye uptake onto raw coal, raw coal with MNBs, SC and SC with MNBs.

Conditions	Pseudo-First-Order			Pseudo-Second-Order		
	q_e1_	K_1_	R^2^	q_e2_	K_2_	R^2^
SC	6.8295	0.0087	0.9080	15.6519	0.0031	0.9911
SC with MNBs	2.2769	0.0090	0.8106	16.1447	0.0144	0.9996
Raw	3.0155	0.0167	0.9288	9.1794	0.0152	0.9996
Raw with MNBs	1.8687	0.0163	0.9040	12.3655	0.0275	0.9999

**Table 2:** Langmuir and Freundlich thermodynamic fitting parameters for raw coal, raw coal with MNBs, SC and SC with MNBs.

T/°C	Condition	Langmuir Model			Freundlich Model		
		b	q_0_(mg/g)	R^2^	n	k	R^2^
25	Raw coal	0.0133	15.7530	0.9970	2.5649	1.4337	0.9637
	Raw coal with MNBs	0.0057	36.6435	0.9966	1.7014	0.8533	0.9879
	SC	0.0028	63.4518	0.9552	1.3854	0.4847	0.9955
	SC with MNBs	0.0010	169.4915	0.8781	1.1444	0.2741	0.9981
35	Raw coal	0.0127	16.8606	0.9930	2.4592	1.3881	0.9432
	Raw coal with MNBs	0.0047	43.4972	0.9970	1.5827	0.7335	0.9875
	SC	0.0022	77.5795	0.9262	1.3217	0.4148	0.9996
	SC with MNBs	0.0008	201.6129	0.8573	1.1229	0.2566	0.9988

**Table 3:** Comparison of SC with MNBs and references.

Adsorbent	Time (min)	Adsorption Capacity (mg/g)	Refs.
Chi-Fe-Pd-Ir	60	93.4	[51]
ASL	720	293.26	[52]
HTN-CS	90	374.4	[53]
Zn(cur)O NPs	110	104.91	[54]
Zn(Cur)O	110	89.85	[55]
SC with MNBs	20	169.49	This work

## Data Availability

Not applicable.

## Sample Availability

Not available.
